# Predictors of survival and functional outcomes in natalizumab-associated progressive multifocal leukoencephalopathy

**DOI:** 10.1007/s13365-015-0316-4

**Published:** 2015-03-14

**Authors:** Tuan Dong-Si, Sarah Gheuens, Amy Gangadharan, Made Wenten, Jeffrey Philip, James McIninch, Shoibal Datta, Nancy Richert, Carmen Bozic, Gary Bloomgren, Sandra Richman, Thomas Weber, David B. Clifford

**Affiliations:** 1 Drug Safety and Risk Management, Biogen Idec Inc., 225 Binney Street, Cambridge, MA 02142 USA; 2 Data Sciences, Biogen Idec Inc., Cambridge, MA USA; 3 Neurology Research and Development, Biogen Idec Inc., Cambridge, MA USA; 4 Marienkrankenhaus, Academic Teaching Hospital, University of Hamburg, Hamburg, Germany; 5 Department of Neurology, Washington University School of Medicine, Saint Louis, MO USA

**Keywords:** Natalizumab, Progressive multifocal leukoencephalopathy, Expanded Disability Status Scale, Karnofsky Performance Scale, Survival

## Abstract

Natalizumab, a highly effective therapy for relapsing-remitting multiple sclerosis, is associated with a risk of progressive multifocal leukoencephalopathy (PML). The objective of this analysis was to examine factors predicting survival in a large natalizumab-associated PML global population. Patients with natalizumab-associated PML identified through postmarketing surveillance were followed up for up to 24 months using a structured questionnaire completed by treating physicians. Demographic and clinical characteristics, JC viral load, magnetic resonance imaging (MRI) results, and Expanded Disability Status Scale (EDSS) and Karnofsky Performance Scale (KPS) scores were compared in survivors and nonsurvivors. Kaplan-Meier analysis was used to model survival function. Among the 336 patients included in this analysis, 76 % survived, with mean follow-up time from PML diagnosis of 16.1 months for survivors; mean time from diagnosis to death was 4.7 months for nonsurvivors. Survivors were significantly younger at diagnosis, had significantly lower EDSS scores and higher KPS scores prior to PML diagnosis, and had significantly lower cerebrospinal fluid JC viral load at the time of diagnosis. Patients with less extensive disease on MRI at diagnosis had a higher survival rate than those with widespread disease. Survivors generally had less functional disability pre-PML, at PML diagnosis, and in subsequent months. In survivors, functional disability appeared to stabilize approximately 6 months post-PML diagnosis. In this analysis, younger age at diagnosis, less functional disability prior to PML diagnosis, lower JC viral load at diagnosis, and more localized brain involvement by MRI at the time of diagnosis appeared to predict improved survival in natalizumab-associated PML.

**Electronic supplementary material:**

The online version of this article (doi:10.1007/s13365-015-0316-4) contains supplementary material, which is available to authorized users.

## Introduction

Multiple sclerosis (MS) is an immune-mediated disease resulting in progressive neurological disability. Natalizumab reduces the rate of clinical relapse and improves disability status in patients with relapsing forms of the disease (Polman et al. 2006; Belachew et al. 2011). Natalizumab is also associated with progressive multifocal leukoencephalopathy (PML), an opportunistic demyelinating disease of the brain caused by the JC virus (JCV) (Langer-Gould et al. 2005). In patients who test positive for anti-JCV antibodies, the risk of natalizumab-associated PML increases with treatment duration (especially >2 years of therapy) and prior use of immunosuppressant therapy (Bloomgren et al. 2012).

Although highly variable, survival in patients with acquired immune deficiency syndrome (AIDS)-associated PML has been linked to specific patient characteristics, JC viral load in cerebrospinal fluid (CSF) (Yiannoutsos et al. 1999), and underlying immunologic function, such as low CD4+ cell count at disease onset (Gasnault et al. 2011).

Few data exist regarding prognostic markers of natalizumab-associated PML. An analysis of the first 35 PML patients concluded that survivors were younger, had a lower Kurtzke Expanded Disability Status Scale (EDSS) (Kurtzke 1983) score prior to PML, had a shorter duration of symptom onset to PML diagnosis, and typically had magnetic resonance imaging (MRI) findings consistent with more localized disease than patients who did not survive (Vermersch et al. 2011). However, only 12 PML survivors had physician-reported disability scores (based on the Karnofsky Performance Scale [KPS] [Karnofsky and Burchenal 1949]) available beyond 6 months following PML diagnosis. The small sample size and lack of available follow-up data limited longitudinal extrapolation of these results.

## Patients and methods

As of August 22, 2013, there were 398 confirmed cases of natalizumab-associated PML globally, all spontaneously reported in the postmarketing setting.

In this study, the diagnosis of PML was confirmed by one of two sets of criteria:

1. Brain tissue (from biopsy or postmortem examination) showing evidence of viral cytopathic changes on hematoxylin and eosin staining associated with either positive immunohistochemistry for SV40 or in situ hybridization for JCV DNA.
2. PCR detection of JCV DNA in CSF or in brain biopsy specimens, preferably by ultrasensitive quantitative PCR testing (limit of quantification of ≤50 copies/mL), along with a detailed description of brain MRI findings consistent with PML (Dong-Si et al. 2012).

Treating physicians were queried at the time of Biogen Idec case confirmation and every 6 months for up to 24 months using a PML Data Collection Tool (DCT) (Supplementary Fig. 1) designed to solicit information regarding a patient’s PML disease status, survival status, clinical characteristics (e.g., functional disability using the EDSS [Kurtzke 1983; Supplementary Table 1a] and KPS [Karnofsky and Burchenal 1949; Supplementary Table 1b]), and additional pertinent data (e.g., demographics, natalizumab exposure, and prior immunosuppressant use). Because this was an analysis of aggregate anonymous data collected from reports of serious adverse events, neither ethics committee approval nor informed consent was required. Patient data were supplemented by information captured in the Biogen Idec natalizumab global safety database. PML patients were categorized by survival status as of the data cutoff date (August 22, 2013). Survivors were defined as patients who were alive at the data cutoff date and had follow-up data for at least 6 months after PML diagnosis; nonsurvivors were defined as patients who died at any time after PML diagnosis. JC viral load (copies/mL) at time of PML diagnosis was defined as the patient’s earliest positive quantitative value in a CSF sample (an average value was calculated if multiple results originated on the same date). Qualitative JC viral load results without quantification were excluded. EDSS and KPS data for survivors were analyzed at the following six time points: on natalizumab pre-PML diagnosis (the earliest available score); at PML diagnosis; and four intervals post-PML diagnosis (6–9, 10–13, 14–17, and ≥18 months). Average scores were calculated if multiple scores were available within given time intervals. Treating physicians provided MRI data from the time of PML diagnosis. Information regarding date and cause of death was collected whenever possible.

### Statistical analyses

#### Demographics and predictors of survival

We evaluated differences in demographic and clinical characteristics in survivors and nonsurvivors, overall and stratified by geography. Survival rates were stratified by PML case numbers, which were assigned chronologically (1–100, 101–200, and 201–300).

Categorical variables were presented as frequencies and compared using chi-square tests. Continuous variables were assessed for normality using the Shapiro-Wilk test. Age was the only variable that met the normality assumption, and it was compared using an independent *t* test. The Mann-Whitney-Wilcoxon two-sample test was used to test for differences in continuous variables that did not meet the normality assumption. All tests assumed a two-sided alternative hypothesis and a 0.05 significance level uncorrected for multiple comparisons. All analyses were conducted using SAS/STAT® software, version 9.3, and R (R Development Core Team 2014).

#### EDSS and KPS analysis

A weighted polynomial regression model, employing the locally weighted scatterplot smoothing (LOWESS) algorithm (Cleveland 1981), was used to assess EDSS and KPS data and to derive functional outcome by survival status. The LOWESS curve is a nonparametric regression method that combines multiple regression models in a k-nearest-neighbor-based meta-model. Its use is recommended in cases where there is no a priori model to which scatter data can be fit and works by creating a fit function based on localized subsets of the data. A curve fitting the localized models is smoothed by a locally weighted, linear, least squares method. Here, we use the implementation of LOWESS provided in the core R language package, “stats” (R Development Core Team 2014).

#### Time from PML diagnosis to death

A Kaplan-Meier estimator of the survival function was generated to model patient vital status. The Kaplan-Meier estimator is often used in medical research to measure the fraction of patients living for a certain amount of time after treatment (Kaplan and Meier 1958). Here, the survival function is based on the fraction of patients living for a certain amount of time after PML diagnosis. We used the Kaplan-Meier estimator implementation provided in the R-language package, “survival” (Therneau 2013). The model uses type I right-censoring of data, with survivorship defined in terms of time interval from date of diagnosis to data cutoff.

## Results

### Patient demographics and survival

Of the 398 natalizumab-treated patients with PML identified as of August 22, 2013, 62 patients were alive but had not reached the 6-month time point after PML diagnosis (early PML). The remaining 336 patients were alive for at least 6 months after PML diagnosis or had died and therefore were included in this evaluation. At the time of this analysis, there were 254 survivors (76 %) and 82 nonsurvivors (24 %). For the 254 survivors, the mean (median) follow-up time from PML diagnosis was 16.1 months (17.2 months).

### Predictors of survival

Compared with nonsurvivors, survivors were younger, had better pre-PML EDSS (mean 3.7 and median 3.5 vs mean and median 5.0; *p* = 0.0028) and KPS scores (mean 81.2 and median 80 vs mean 70.8 and median 70; *p* = 0.0117), and had lower JC viral load in the CSF at the time of PML diagnosis (Table 1). After PML diagnosis, corticosteroid (CS) use was not different between survivors (78.3 %) and nonsurvivors (75.6 %).

**Table 1:** Patient demographics and baseline characteristics

Characteristic	All (*N* = 336)	Survivors (*n* = 254)	Nonsurvivors (*n* = 82)	*p* value
Age at diagnosis, years	(*n* = 332)	(*n* = 252)	(*n* = 80)	
Mean (SD)	45.0 (9.6)	43.5 (9.2)	49.5 (9.7)	<0.0001
Median (range)	45 (15–73)	44 (15–71)	50 (24–73)	
Gender, female, *n* (%)	237 (71)	182 (72)	55 (67)	0.4322
Geography, *n* (%)				
USA	119 (35)	70 (59)	49 (41)	<0.0001^b^
EU/ROW	217 (65)	184 (85)	33 (15)	
Duration of MS, years	(n = 116)	(n = 90)	(n = 26)	
Mean (SD)	14.1 (8.2)	13.3 (7.7)	16.7 (9.1)	0.0909
Median (range)	12 (1–51)	12 (1–51)	15 (6–38)	
Natalizumab exposure, months				
Mean (SD)	38.6 (14.0)	39.2 (14.0)	36.7 (13.8)	0.1045
Median (range)	38 (8–74)	40 (8–74)	34 (14–72)	
JC viral load, copies/mL	(n = 285)	(n = 216)	(n = 69)	
Mean (SD)	185,797 (893,482)	91,587 (469,668)	480,715 (1,587,521)	<0.0001
Median (range)	500 (1–10,243,280)	386 (1–4,831,575)	2076 (10–10,243,280)	
Time from symptom onset to diagnosis, days	(n = 328)	(n = 246)	(n = 82)	
Mean (SD)	44.2 (51.9)	41.3 (44.5)	52.9 (69.2)	0.2623
Median (range)	27 (0–368)	26 (0–216)	29 (0–368)	
EDSS score, pre-PML	(n = 123)	(n = 101)	(n = 22)	
Mean (SD)	3.9 (1.8)	3.7 (1.8)	5.0 (1.7)	0.0028
Median (range)	3.75 (0–8)	3.5 (0–7)	5.0 (2–8)	
KPS score, pre-PML	(n = 84)	(n = 72)	(n = 12)	
Mean (SD)	79.7 (13.2)	81.2 (12.4)	70.8 (15.1)	0.0117
Median (range)	80 (40–100)	80 (40–100)	70 (40–100)	
Prior immunosuppressant use^a^, *n* (%)	91 (27)	68 (27)	23 (28)	

^a^Mitoxantrone, methotrexate, azathioprine, cyclophosphamide, or mycophenolate

^b^ *p* value is for comparison of survival rate between USA and EU/ROW. All other *p* values are for comparison between survivors and nonsurvivors

*SD* standard deviation

Compared with patients from the USA, a greater proportion of patients from Europe (EU) or the rest of the world (ROW) were survivors (59 vs 85 %; *p* < 0.0001). For the first 100 PML cases (confirmed between August 2008 and March 2011), the survival rate was 49 % in the USA and 85 % in the EU/ROW. This geographical difference became smaller over time: USA 58 % and EU/ROW 85 % for the next 100 cases (confirmed between March 2011 and January 2012); USA 74 % and EU/ROW 81 % for the next 100 cases (confirmed between January 2012 and October 2012). US patients were generally older than EU/ROW patients, regardless of the chronologically assigned case number (Supplementary Table 2); the mean (median) age in the USA was 53 years (55 years) in nonsurvivors and 47 years (47 years) in survivors, while the mean (median) age in the EU/ROW was 45 years (46 years) in nonsurvivors and 42 years (42 years) in survivors. There was no difference in prior IS use or pre-PML EDSS and KPS scores stratified by geographies (data not shown).

### JC viral load adjusted for age

**Fig. 1:**
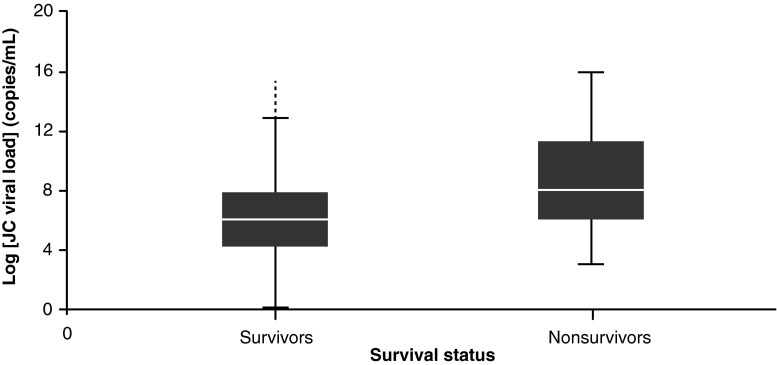
Log JC viral load (copies/mL) for PML patients according to survival status (*p* < 0.0001). *p* value adjusted for age. *White horizontal line*, median; *horizontal bars*, range

Age at PML diagnosis was a potential confounder (*r* = 0.0187; *p* = 0.0035) in the relationship between JC viral load and outcome (survivor/nonsurvivor). Therefore, JC viral load was adjusted for age in the regression using a log transformation of JC viral load, with outcome and age as the independent variables (Fig. 1). Age remained a significant predictor of survival (*p* < 0.0001).

### JC viral load in relation to MRI at diagnosis

Brain MRI results at PML diagnosis were available for 296 patients, including 223 survivors and 73 nonsurvivors (Table 2). A higher survival rate was seen in patients with less extensive disease on MRI at diagnosis (i.e., unilobar PML) than in those with widespread disease (79 vs 66 %; Table 2). Of those patients with less extensive PML lesions (*n* = 115), the majority (52 %) had frontal lobe lesions, 29 % had occipital or parietal lobe lesions, 3 % had temporal lobe lesions, 3 % had lesions in the basal ganglia/thalamus, and the remaining cases were unreported.

**Table 2:** MRI findings at diagnosis of PML

PML extension at diagnosis	All (*N* = 296)	Survivors (*n* = 223)	Nonsurvivors (*n* = 73)	Survival (%)
Unilobar, *n* (%)^a^	115 (39)	91 (41)	24 (33)	79
Widespread, *n* (%)^b^	108 (36)	71 (32)	37 (51)	66

^a^Unilobar lesions were confined to one lobe

^b^Widespread lesions involved two or more noncontiguous lobes and/or lesions present in both hemispheres

For both unilobar and widespread disease, JC viral load at PML diagnosis was significantly lower in survivors than in nonsurvivors (unilobar, median 275 copies/mL vs median 2903 copies/mL, *p* = 0.0157; widespread, median 416 copies/mL vs median 1631 copies/mL, *p* = 0.0014) (Supplementary Table 3).

### Functional outcomes of survivors

#### EDSS scores

**Fig. 2:**
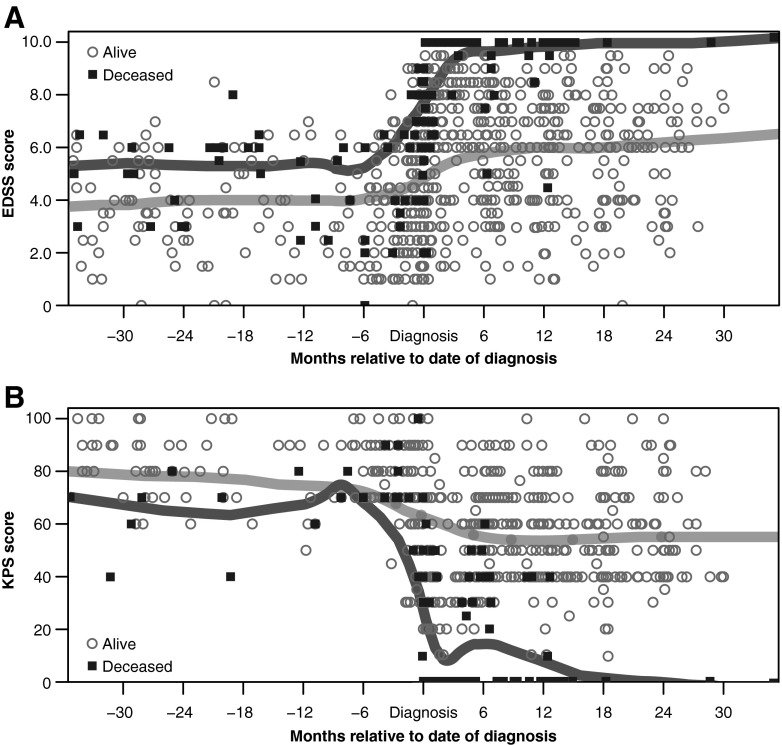
Functional outcomes as measured by **a** EDSS and **b** KPS scores for PML patients according to survival status. *Each symbol* represents a single patient measurement at a single time point. *The light gray lines* represent polynomial regression trend lines (LOWESS curves) for survivors; *the dark gray lines* represent polynomial regression trend lines (LOWESS curves) for nonsurvivors. EDSS and KPS scores were not available for all patients at all time points

At least one EDSS score was available for 194 PML survivors and 77 nonsurvivors (Fig. 2a). Mean EDSS scores showed little variation in the months prior to PML diagnosis, when patients were receiving natalizumab. In general, PML patients who survived had lower mean pre-PML EDSS scores than nonsurvivors; this trend was maintained at PML diagnosis and following diagnosis. Mean EDSS scores for both patient groups increased shortly after diagnosis, with a more marked increase in functional disability observed in nonsurvivors (Fig. 2a). Thereafter, mean EDSS scores appeared to plateau, and in survivors, scores appeared to stabilize at approximately 6 months post-PML diagnosis.

#### KPS scores

At least one KPS score was available for 191 PML survivors and 77 nonsurvivors (Fig. 2b). In the months prior to PML diagnosis, when patients were receiving natalizumab, mean KPS scores showed little variation for both survivors and nonsurvivors. The decrease in KPS scores observed shortly after PML diagnosis, which indicated an increase in functional disability, was more pronounced in nonsurvivors. Mean KPS scores, particularly for the surviving patients, appeared to remain stable at all subsequent time points.

#### Correlation between EDSS and KPS scores

To assess the correlation between measures of functional disability, a weighted polynomial regression model was applied, as displayed in Supplementary Fig. 2. The EDSS and KPS were negatively correlated (*R* ^2^ = 0.713) in survivors.

### Time from PML diagnosis to death and cause of death

**Fig. 3:**
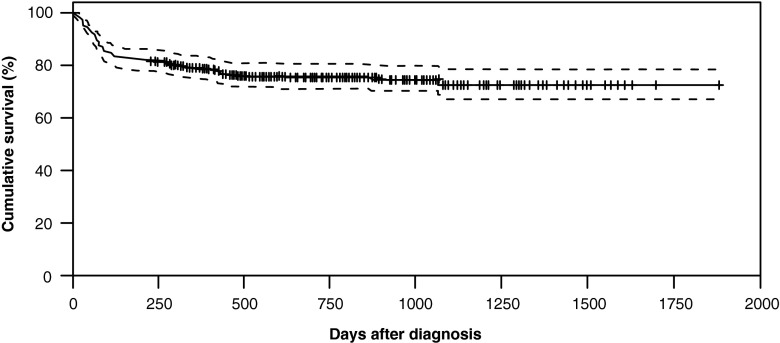
Kaplan-Meier estimate of overall survival after PML diagnosis

Date of death was unknown for three patients with fatal outcome. For the remaining 79 patients with fatal outcome, the mean time from date of PML diagnosis to death was 4.7 months. Fifty-nine patients (74.7 %) died within 6 months of PML diagnosis. Overall survival of the PML population plateaued around 4–6 months (Fig. 3).

Cause of death was unknown for 53 patients; for the remaining patients, the cause of death was pneumonia for 11; cardiorespiratory event for 6; dehydration and nonfeeding for 4; mass effect/edema for 3; and palliative sedation, suicide, seizure, sepsis, and urosepsis for 1 patient each. Hospice care was mentioned for 25 patients, 9 of whom died more than 6 months after PML diagnosis.

## Discussion

Although PML was once considered uniformly fatal, our analysis of the first 336 confirmed postmarketing cases of natalizumab-associated PML with up to 24 months of follow-up data revealed a 76 % survival rate over the study period. In contrast, survival at 1 year with PML in other disease populations has been reported to vary from less than 20 to 58 %, depending on the underlying condition (De Luca et al. 1998; Marzocchetti et al. 2009; Hall et al. 1998). Recent observations suggest an improved 1-year survival rate of 75 % in HIV+ patients who were treated early after disease onset aggressively with effective anti-HIV therapy, resulting in prompt restoration of CD4+ counts and inhibition of HIV replication (Gasnault et al. 2011; Fanjul et al. 2013). In contrast to natalizumab-associated PML, HIV+ PML appears to show a longer survival with increasing age (Casado et al. 2013), while age does not seem to influence survival in transplant recipients with PML (Mateen et al. 2011).

Unlike most other patients with PML, MS patients with natalizumab-associated PML have a functioning systemic immune system, and therapeutic immune reconstitution may be rapidly achieved through plasma exchange (Gheuens et al. 2013). Although clinical deterioration in the setting of immune reconstitution inflammatory syndrome (IRIS) occurs transiently, without any known effective JCV therapy, this essential therapeutic step is routinely possible in natalizumab-treated patients. It is notable that there appeared to be a learning effect in the current study, with survival rates improving over time in the USA, potentially due to improved detection and management of PML once diagnosed (Giacomini et al. 2014).

Our analysis identified several predictors of survival in natalizumab-associated PML. Younger patients and patients with less disability prior to PML diagnosis were more likely to survive, consistent with previous observations (Vermersch et al. 2011). Although more recent PML cases exhibited fewer geographical differences in survival, age appeared to be an important contributor to overall survival when it was stratified by geography, with US cases being older than ROW cases (median [range] age 55 years [32 − 73 years] in the USA and 46 years [30 − 59 years] in ROW). Older age as a risk factor for poorer outcomes in PML might be important for prescribers to consider in discussions about PML risk.

The criteria for PML diagnosis used in this study allowed for the inclusion of PML patients who were diagnosed, with or without symptoms, on the basis of PCR detection of JCV DNA in CSF or in brain biopsy specimens and a detailed description of brain MRI findings consistent with PML. It is possible that the means of PML diagnosis may have affected outcomes. In a recently published related analysis, patients who were asymptomatic when they were diagnosed with PML had better survival and functional outcomes than patients who were symptomatic at diagnosis. The average time to PML diagnosis was shorter in asymptomatic patients than in symptomatic patients (12 vs 28 days); earlier diagnosis in asymptomatic patients may have allowed for earlier intervention, which may have led to better outcomes (Dong-Si et al. 2014).

The correlation of disease burden and JC viral load in CSF is modest, but our findings show that more advanced disease both by greater MRI lesion burden as well as JC viral load predicts poorer outcomes. Similarly, in a study of 28 HIV+ and HIV− PML patients, those with a higher JC viral load in the CSF at the time of PML diagnosis experienced poorer outcomes (Gasnault et al. 2011). These findings may be driven by fast clearance of the virus in the setting of immune reconstitution, which is probably easier to obtain when there is still localized disease or a lesser amount of antigen. Patients who survive are then able to clear the virus from the CNS (Gasnault et al. 2011; Delbue et al. 2012). While the association between JC viral load in the CSF and survival was statistically significant in our study, this finding should be interpreted with caution, as the association was weak and the relevance of viral load in the CSF—which is not the site of clinically meaningful disease—is unknown.

We did not observe a difference in CS use between survivors and nonsurvivors. CS might enhance survival by blocking overly aggressive IRIS or might inhibit immune control of virus by decreasing the frequency of interferon-gamma-producing JCV-specific CD8+ T cells (Antoniol et al. 2012). This specific T cell response is considered important in PML survival (Gheuens et al. 2011). The proportion of patients with prior use of immunosuppressants did not appear to differ between survivors and nonsurvivors in this study. While prior immunosuppressant use is a known risk factor for development of natalizumab-associated PML, it is not known to be related to PML survival.

Most deaths in our study occurred within the first 6 months after PML diagnosis, consistent with the subacute nature of PML. As survival appears to stabilize at 15 months, it is unlikely that the later deaths occurred as a direct consequence of PML, which is consistent with previous observations (Marzocchetti et al. 2009; Lima et al. 2010). Higher mortality in the early era of natalizumab-associated PML may have resulted from assessment of the prognosis as hopeless and abandonment of supportive care. Experience suggests that supportive care and treatment of IRIS allow survival and in some cases significant functional recovery.

Functional disability stabilized at 6 months post-PML diagnosis and remained relatively stable even beyond 18 months post-PML diagnosis. Varying levels of disability (mild to severe) were present at all studied time points. While not all patients returned to their pre-PML level of disability, the majority of patients appeared to sustain moderate to moderate-severe disability throughout the course of PML and recovery.

Although the EDSS score is designed for MS (Kurtzke 1983) and is commonly used as a functional disability measure, it can be complicated to calculate and has been criticized for relying heavily on walking as the main measure of disability (Amato and Portaccio 2007). The KPS is not specific for MS or PML. It can be used across many diseases, and scores are easier to assess, using an 11-point scale (Karnofsky and Burchenal 1949). EDSS and KPS are highly correlated (Supplementary Fig. 2), and study results show a higher risk of mortality in PML if disability is severe prior to treatment. Clinicians may find this helpful in discussions with patients with advanced MS who are considering the use of natalizumab.

Our study has several limitations. Most importantly, data sets gathered spontaneously in the postmarketing setting are seldom complete, and this might have introduced an unknown bias. Another potential source of bias is the exclusion of PML patients who were alive at the time of this analysis but had not survived for at least 6 months from PML diagnosis. Although immune reconstitution is important, we were not able to reliably evaluate the impact of IRIS or differences in how IRIS may have been managed across this population. In addition, we were not able to take into account lesion volume or specific lesion location. Although there are conflicting results regarding lesion location and prognosis (Post et al. 1999), lesions might be worse in certain locations, for instance, the cerebellum, than in others (Lima et al. 2010).

This is the largest study providing longitudinal follow-up of survival and functional outcomes of natalizumab-associated PML. Age, JCV viral load in CSF, and extent of disease on imaging are predictors of survival. Patients quickly stabilize after the initial phase of disease, and there is a high correlation between EDSS and KPS scores, the latter being more easily obtainable. These predicting factors could be useful when considering treatment options for patients and when counseling them regarding the benefits and risks of treatment (Rudick 2011).

## Electronic supplementary material

Below is the link to the electronic supplementary material.

ESM 1 (DOC 130 kb)

ESM 2 (DOC 149 kb)

ESM 3 (DOC 63 kb)

ESM 4 (DOC 52 kb)

ESM 5 (DOC 47 kb)
