# Supplementary material for: Predictors of survival and functional outcomes in natalizumab-associated progressive multifocal leukoencephalopathy
Source: J Neurovirol. 2015 Mar 14;21(6):637–44. doi: 10.1007/s13365-015-0316-4 (PMC4628054; doi:10.1007/s13365-015-0316-4)
Supplement: Supplementary file 1 — (DOC 130 kb) [file 13365_2015_316_MOESM1_ESM.doc]

**Supplementary Fig. 1** Retrospective and prospective data collection

**
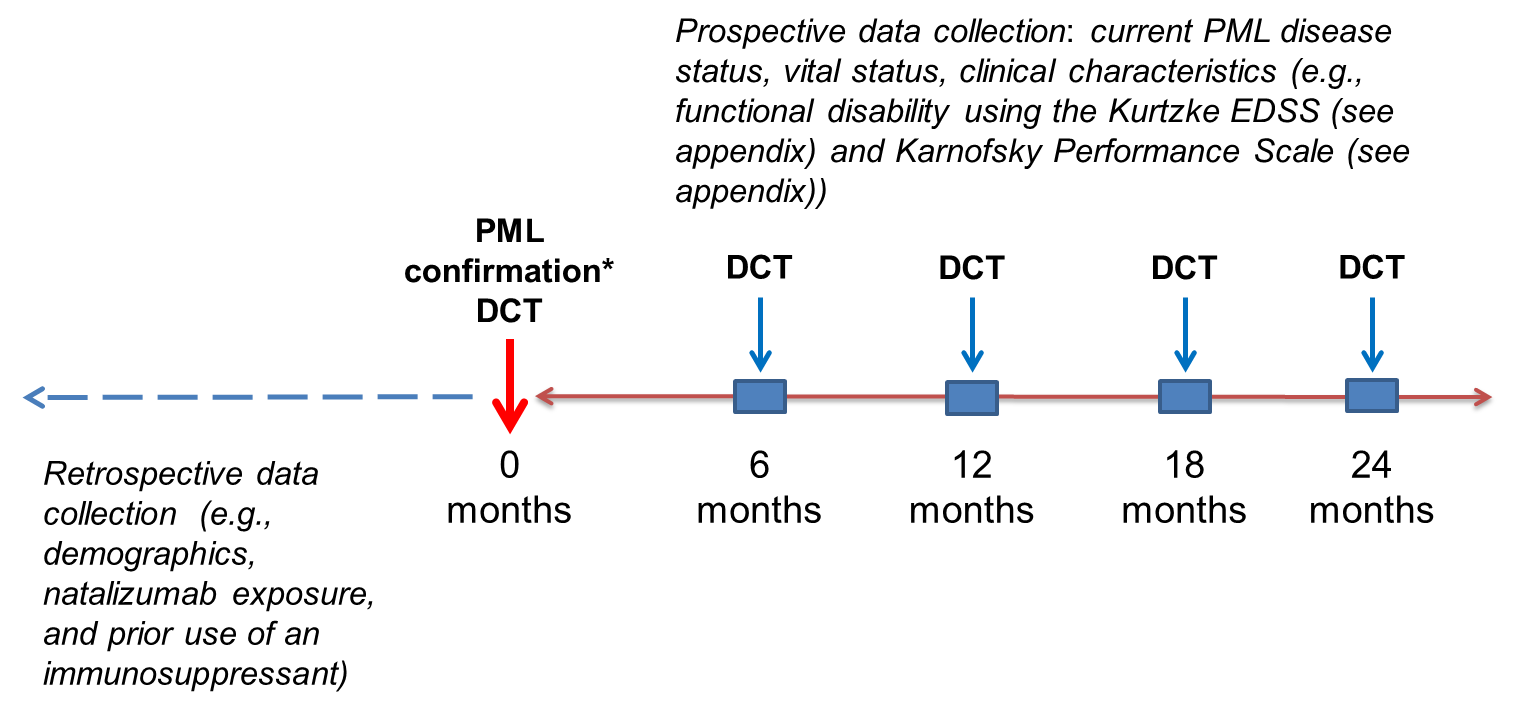
**

*Prospective data collection: current PML disease status, vital status, clinical characteristics (e.g. functional disability using the EDSS and KPS (see Supplementary Table 1)*

*Retrospective data collection (e.g., demographics, natalizumab exposure, and prior use of an immunosuppressant)*

*Date of Biogen Idec case confirmation

EDSS, Expanded Disability Status Scale; KPS, Karnofsky Performance Scale; DCT, Data Collection Tool administration (see Patients and Methods section)
