# Supplementary material for: Predictors of survival and functional outcomes in natalizumab-associated progressive multifocal leukoencephalopathy
Source: J Neurovirol. 2015 Mar 14;21(6):637–44. doi: 10.1007/s13365-015-0316-4 (PMC4628054; doi:10.1007/s13365-015-0316-4)
Supplement: Supplementary file 2 — (DOC 149 kb) [file 13365_2015_316_MOESM2_ESM.doc]

**Supplementary Fig. 2** EDSS and KPS score correlation for surviving patients


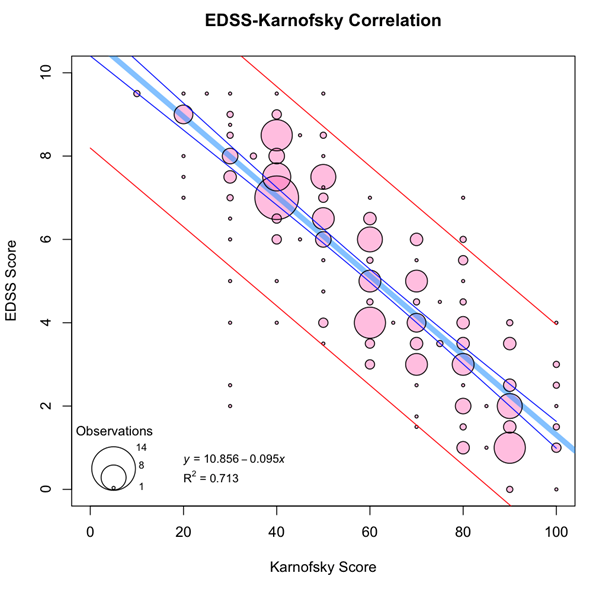


The diameter of the bubbles is proportional to the number of assessments where the indicated EDSS and Karnofsky scores were reported together. The heavy light-blue line represents a linear regression fit to the data. The thin blue lines represent the 95% model confidence boundary. The thin red lines represent the 95% prediction confidence interval, depicting that the model predicts that 95% of observations will occur within those bounds. The fit line is [EDSS] = 10.8322 – 0.095 * [Karnofsky]. The range of the 95% predictor confidence boundary is ±2.659544. EDSS, Expanded Disability Status Scale; KPS, Karnofsky Performance Scale
