# Supplementary material for: Predictors of survival and functional outcomes in natalizumab-associated progressive multifocal leukoencephalopathy
Source: J Neurovirol. 2015 Mar 14;21(6):637–44. doi: 10.1007/s13365-015-0316-4 (PMC4628054; doi:10.1007/s13365-015-0316-4)
Supplement: Supplementary file 3 — (DOC 63 kb) [file 13365_2015_316_MOESM3_ESM.doc]

**Supplementary Table 1** Measures of functional disability (Kurtzke 1983; Karnofksy and Burchenal 1949)

**A Kurtzke Expanded Disability Status Scale (EDSS)**

| **Score** | **Description** |
| --- | --- |
| **0.0** | Normal neurological exam |
| **1.0** | No disability, minimal signs in one functional system (FS) |
| **1.5** | No disability, minimal signs in more than one FS |
| **2.0** | Minimal disability in one FS |
| **2.5** | Mild disability in one FS or minimal disability in two FS |
| **3.0** | Moderate disability in one FS, or mild disability in three or four FS. No impairment to walking |
| **3.5** | Moderate disability in one FS and more than minimal disability in several others. No impairment to walking |
| **4.0** | Significant disability but self-sufficient and up and about some 12 hours a day. Able to walk without aid or rest for 500 meters |
| **4.5** | Significant disability but up and about much of the day, able to work a full day, may otherwise have some limitation of full activity or require minimal assistance. Able to walk without aid or rest for 300 meters |
| **5.0** | Disability severe enough to impair full daily activities and ability to work a full day without special provisions. Able to walk without aid or rest for 200 meters |
| **5.5** | Disability severe enough to preclude full daily activities. Able to walk without aid or rest for 100 meters |
| **6.0** | Requires a walking aid—cane, crutch, etc—to walk about 100 meters with or without resting |
| **6.5** | Requires two walking aids—pair of canes, crutches, etc—to walk about 20 meters without resting |
| **7.0** | Unable to walk beyond approximately 5 meters even with aid. Essentially restricted to wheelchair, though wheels self in standard wheelchair and transfers alone. Up and about in wheelchair some 12 hours a day |
| **7.5** | Unable to take more than a few steps. Restricted to wheelchair and may need aid in transferring. Can wheel self but cannot carry on in standard wheelchair for a full day and may require a motorised wheelchair |
| **8.0** | Essentially restricted to bed or chair or pushed in wheelchair. May be out of bed itself much of the day. Retains many self-care functions. Generally has effective use of arms |
| **8.5** | Essentially restricted to bed much of day. Has some effective use of arms, retains some self-care functions |
| **9.0** | Confined to bed. Can still communicate and eat |
| **9.5** | Confined to bed and totally dependent. Unable to communicate effectively or eat/swallow |
| **10.0** | Death due to MS |

**B Karnofsky Performance Scale (KPS)**

| **Mild**  Able to carry on normal activity and to work; no special care needed | 100 | Normal; no complaints; no evidence of disease |
| --- | --- | --- |
|  | 90 | Able to carry on normal activity; minor signs or symptoms of disease |
|  | 80 | Normal activity with effort; some signs or symptoms of disease |
| **Moderate**  Unable to work; able to live at home and care for most personal needs; varying amount of assistance needed | 70 | Cares for self; unable to carry on normal activity or to do active work |
|  | 60 | Requires occasional assistance, but is able to care for most of his personal needs |
|  | 50 | Requires considerable assistance and frequent medical care |
| **Severe**  Unable to care for self; requires equivalent of institutional or hospital care; disease may be progressing rapidly | 40 | Disabled; requires special care and assistance |
|  | 30 | Severely disabled; hospital admission is indicated although death not imminent |
|  | 20 | Very sick; hospital admission necessary; active supportive treatment necessary |
|  | 10 | Moribund; fatal processes progressing rapidly |
|  | 0 | Dead |
