# Supplementary material for: Predictors of survival and functional outcomes in natalizumab-associated progressive multifocal leukoencephalopathy
Source: J Neurovirol. 2015 Mar 14;21(6):637–44. doi: 10.1007/s13365-015-0316-4 (PMC4628054; doi:10.1007/s13365-015-0316-4)
Supplement: Supplementary file 4 — (DOC 52 kb) [file 13365_2015_316_MOESM4_ESM.doc]

**Supplementary Table 2** Patient age at diagnosis of PML stratified by geography, survival status, and chronologically assigned PML case number

| **Patient case number** | **US** | | **Europe/Rest of world** | |
| --- | --- | --- | --- | --- |
|  | **Nonsurvivors (n=49)** | **Survivors (n=70)** | **Nonsurvivors (n=33)** | **Survivors (n=184)** |
| All, n  Age at PML diagnosis, years  Mean  Median (range) | 49  53  55 (32−73) | 70  47  47 (23−71) | 31  45  46 (30−59) | 182  42  42 (15−62) |
| 0−100 (Aug 2008−Mar 2011), n  Age at PML diagnosis, years  Mean  Median (range) | 21  50  52 (33−67) | 20  45  43 (23−67) | 9  47  49 (36−57) | 50  42  42 (27−61) |
| 101−200 (Mar 2011−Jan 2012), n  Age at PML diagnosis, years  Mean  Median (range) | 14  56  57 (52−61) | 19  47  48 (26−71) | 11  44  43 (39−53) | 56  43^a^  42 (15−61) |
| 201−300 (Jan 2012−Oct 2012), n  Age at PML diagnosis, years  Mean  Median (range) | 9  52  54 (32−73) | 22  50  49 (30−64) | 13  44^b^  46 (46−59) | 56  43^a^  43 (22−62) |

**^a^**Age unknown for one case

^b^Unknown for two cases
