# Supplementary material for: Predictors of survival and functional outcomes in natalizumab-associated progressive multifocal leukoencephalopathy
Source: J Neurovirol. 2015 Mar 14;21(6):637–44. doi: 10.1007/s13365-015-0316-4 (PMC4628054; doi:10.1007/s13365-015-0316-4)
Supplement: Supplementary file 5 — (DOC 47 kb) [file 13365_2015_316_MOESM5_ESM.doc]

**Supplementary Table 3** JC viral load (copies/ml) in relation to MRI findings at diagnosis

| **PML extension at diagnosis** | **JC viral load at diagnosis^a^** | | ***p* value** |
| --- | --- | --- | --- |
|  | **Survivors** | **Nonsurvivors** |  |
| Unilobar^b^ | (n=76)  275 | (n=23)  2903 | 0.0157 |
| Widespread^c^ | (n=57)  416 | (n=28)  1631 | 0.0014 |

^a^Median copies/mL

^b^Unilobar lesions were confined to one lobe

^c^Widespread lesions involved two or more noncontiguous lobes and/or lesions present in both hemispheres
